# Prognostic Value of C-Reactive Protein, Glasgow Prognostic Score, and C-Reactive Protein-to-Albumin Ratio in Colorectal Cancer

**DOI:** 10.3389/fcell.2021.637650

**Published:** 2021-10-26

**Authors:** Jiahui Zhou, Wene Wei, Hu Hou, Shufang Ning, Jilin Li, Baoyue Huang, Kaisheng Liu, Litu Zhang

**Affiliations:** ^1^Department of Research, Affiliated Tumor Hospital, Guangxi Medical University, Nanning, China; ^2^Department of Laboratory Medicine, Shenzhen People’s Hospital, The First Affiliated Hospital, Southern University of Science and Technology, Shenzhen, China; ^3^Department of Laboratory Medicine, Shenzhen People’s Hospital, The Second Clinical Medical College, Jinan University, Guangzhou, China; ^4^Guangxi Cancer Molecular Medicine Engineering Research Center, Nanning, China

**Keywords:** colorectal cancer, prognosis, C-reactive protein, Glasgow Prognostic Score, C-reactive protein-to-albumin ratio

## Abstract

**Background:** Emerging evidence suggests that inflammatory response biomarkers are predictive factors that can improve the accuracy of colorectal cancer (CRC) prognoses. We aimed to evaluate the prognostic significance of C-reactive protein (CRP), the Glasgow Prognostic Score (GPS), and the CRP-to-albumin ratio (CAR) in CRC.

**Methods:** Overall, 307 stage I–III CRC patients and 72 colorectal liver metastases (CRLM) patients were enrolled between October 2013 and September 2019. We investigated the correlation between the pretreatment CRP, GPS, and CAR and the clinicopathological characteristics. The Cox proportional hazards model was used for univariate or multivariate analysis to assess potential prognostic factors. A receiver operating characteristic (ROC) curve was constructed to evaluate the predictive value of each prognostic score. We established CRC survival nomograms based on the prognostic scores of inflammation.

**Results:** The optimal cutoff levels for the CAR for overall survival (OS) in all CRC patients, stage I–III CRC patients, and CRLM patients were 0.16, 0.14, and 0.25, respectively. Kaplan–Meier analysis and log-rank tests demonstrated that patients with high CRP, CAR, and GPS had poorer OS in CRC, both in the cohorts of stage I–III patients and CRLM patients. In the different cohorts of CRC patients, the area under the ROC curve (AUC) of these three markers were all high. Multivariate analysis indicated that the location of the primary tumor, pathological differentiation, and pretreatment carcinoembryonic antigen (CEA), CRP, GPS, and CAR were independent prognostic factors for OS in stage I–III patients and that CRP, GPS, and CAR were independent prognostic factors for OS in CRLM patients. The predictors in the prediction nomograms included the pretreatment CRP, GPS, and CAR.

**Conclusions:** CRP, GPS, and CAR have independent prognostic values in patients with CRC. Furthermore, the survival nomograms based on CRP, GPS, and CAR can provide more valuable clinical significance.

## Introduction

Colorectal cancer (CRC) is the third most common cancer and the second most common cause of cancer-related death worldwide. CRC is one of the most common gastrointestinal malignancies (Bray et al., 2018). The liver is the most common metastatic site of CRC, and the most common cause of death associated with CRC is liver metastasis (Hamilton et al., 2014). Surgery is still the primary curative modality for these patients. With increased treatment options, it has become increasingly important to choose the best treatment strategy. However, there is no reliable biomarker to identify stage I–III patients who are at high risk of poor prognosis and need to be treated with adjuvant chemotherapy. Regarding colorectal liver metastases (CRLM), there is a lack of reliable prognostic biomarkers to determine which patients will benefit from chemotherapy. Therefore, to achieve better clinical treatment outcomes, it is necessary to identify biomarkers that can better predict CRC prognoses.

Since Virchow first reported the association between inflammation and cancer in 1863, various studies have analyzed this relationship (Balkwill and Mantovani, 2001; Mantovani et al., 2008). Inflammation has been widely recognized as a factor that contributes to cancer progression, and many inflammatory markers, such as C-reactive protein (CRP), the Glasgow Prognostic Score (GPS), and the modified GPS (mGPS), have been shown to be associated with survival in CRC patients (Nasr et al., 2018; Matsubara et al., 2020). After curative treatment of CRC patients, CRP alone can be used as a strong prognostic indicator (Køstner et al., 2016; Matsubara et al., 2020). The GPS is a simple and valuable cumulative prognostic scoring system. It is based on two simple components—serum CRP and albumin—the levels of which are defined as normal or abnormal based on their laboratory reference ranges. Practitioners can divide cancer patients into three separate groups before treatment based on their GPS (McMillan et al., 2007). The CRP-to-albumin ratio (CAR) is based on a composite ratio. Recently, the value of the CAR in predicting the prognosis of CRC patients has been widely investigated (Ishizuka et al., 2016; Haruki et al., 2017). However, Climent et al. (2019) found that the difference in overall survival (OS) and disease-free survival between the high and low CAR groups was not significant; thus, the prognostic value of the CAR in CRC is still controversial.

Therefore, the present study aimed to assess the relationship between CRP, GPS, and CAR and the prognosis and clinicopathology of patients with CRC and to compare the prognostic value of these indicators in patients undergoing treatment for CRC.

## Patients and Methods

### Patients

**FIGURE 1:**
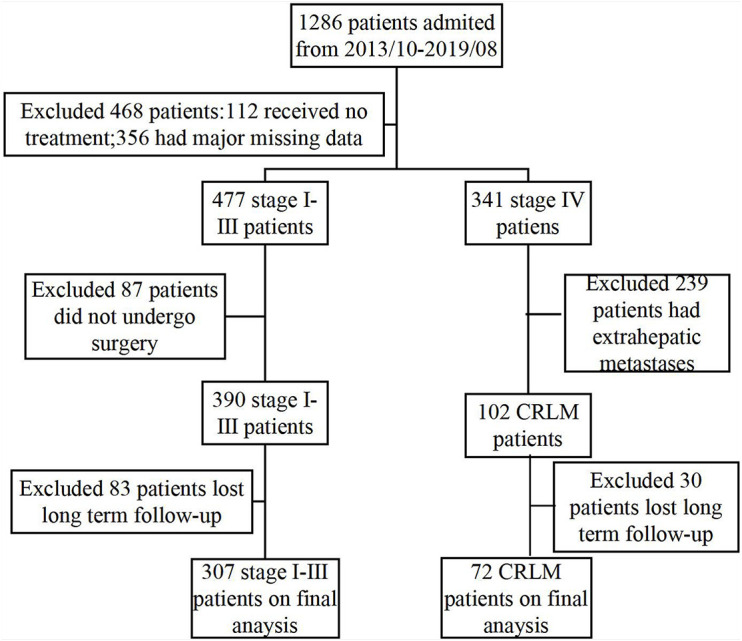
Study design and flowchart.

The data of 1,286 patients with CRC who were admitted to the Gastrointestinal Surgery Department of the Guangxi Medical University Cancer Hospital between October 2013 and September 2019 were retrospectively analyzed. Of these, 379 were included in this study and 907 were excluded because of incomplete medical information or lack of follow-up, the details of which are presented in Figure 1. All patients underwent scanning using abdominal B ultrasound, magnetic resonance imaging, and computed tomography, and they were examined for any clinical symptoms and signs of the disease. The inclusion criteria were as follows: (1) CRC was confirmed either histologically or using imaging techniques and (2) all patients underwent surgical R0 or R1 resection or chemotherapy using leucovorin/fluorouracil/oxaliplatin (FOLFOX)- or Capox-based regimens. The exclusion criteria were as follows: (1) had clinical evidence of infection or other inflammatory diseases; (2) at the time of laboratory testing, anticancer treatments had been administered; and (3) patients with primary tumors in other organs We extracted relevant routine laboratory data measured on the day of admission and the clinicopathological data for the selected CRC patients from electronic medical records, including the serum levels of CRP, albumin, and tumor markers of carcinoembryonic antigen (CEA), carbohydrate antigen 19-9 (CA19-9), age, sex, degree of differentiation, tumor location, and tumor node metastasis stage (Amin et al., 2017). Patients underwent complete follow-up after the initial treatment. The start time of the follow-up was the date of the initial diagnosis of CRC and the end time was August 2020 or date of death.

This study was approved by the Local Ethics Committee of the Guangxi Medical University Cancer Hospital, and it was conducted in accordance with the ethical guidelines of the 2008 Declaration of Helsinki and of the current hospital. All patients provided written informed consent.

### Method for the Detection or Calculation of Each Index

CRP was detected with the latex enhanced immunoturbidimetric method. Albumin was detected using the bromocresol green method. The levels of serum CEA and CA19-9 were measured with a chemiluminescence microparticle immunoassay using the Arhcitect i2000SR analyzer and the corresponding reagent kits, which were purchased from Architect Diagnostics (Abbott Park, IL, United States). All operations followed the manufacturer’s instructions. Systemic inflammatory response was assessed using CRP, GPS, and CAR. GPS is a cumulative score and was calculated as described previously (McMillan, 2013). Patients with both an elevated level of CRP (>10 mg/L) and hypoalbuminemia (albumin < 35 g/L) were categorized as GPS2, and those showing one or neither of these blood chemistry abnormalities were categorized as GPS1 or GPS0, respectively. CAR was calculated as CAR = serum CRP level (mg/L)/serum albumin level (g/L).

### Statistical Analysis

Statistical analyses were conducted using SPSS (version 21.0) and R (version 3.5.1). Data are expressed as the mean ± standard deviation (SD). The optimal cutoff values were assessed by using receiver operating characteristic (ROC) curves based on the Youden index (YI = sensitivity + specificity - 1) and by X-tile software. The end point of survival analysis is OS. Spearman’s or Pearson’s correlation analysis was used to determine the association between CRP, GPS, and CAR and the other clinicopathological features. Kaplan–Meier analysis and log-rank test were used to determine the differences in the survival curves between the two groups. The ROC curve was constructed to evaluate the discriminative ability of each prognostic score, and the area under the ROC curve (AUC) was measured and compared with that obtained by the method established by DeLong et al. (1988). The Cox proportional hazards model was used for univariate and multivariate analyses to assess potential prognostic factors. A nomogram was formulated using R version 3.5.1. The discriminative ability of the nomogram was evaluated with the concordance index (*C*-index). The larger the *C*-index, the more accurate the prognostic prediction. The calibration ability of the nomogram was evaluated by a calibration plot. The closer the calibration curve is to the reference line, the more perfect the calibration. All statistical tests were two-sided, and when the associated probability was less than 0.05, *p*-values were considered statistically significant.

## Results

**FIGURE 2:**
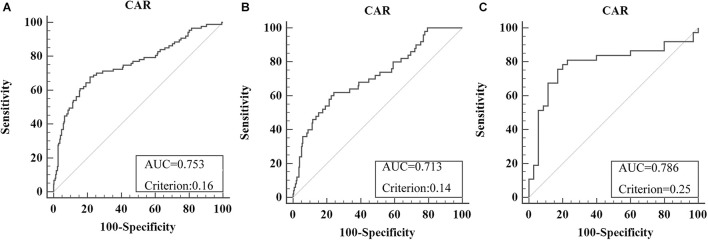
Optimal cutoff levels for the C-reactive protein/albumin ratio (CAR) were applied with the receiver operating characteristic (ROC) curves for overall survival in all recruited colorectal cancer (CRC) patients **(A)**, in stage I–III CRC patients **(B)**, and in colorectal liver metastases (CRLM) patients **(C)**.

Overall, 379 patients were enrolled (male/female = 250:129), including 307 stage I–III patients and 72 CRLM patients. The optimal cutoff level for CAR was 0.16 (AUC = 0.753, sensitivity = 67.82%, specificity = 78.42%) for OS in all recruited CRC patients (Figure 2A). For subgroup analysis, the optimal cutoff level for CAR for OS was 0.14 (AUC = 0.713, sensitivity = 62%, specificity = 75.88%) in all stage I–III CRC patients (Figure 2B), and that in CRLM patients was 0.25 (AUC = 0.786, sensitivity = 75.68%, specificity = 82.86%) (Figure 2C). Using the X-tile software, the optimal cutoff levels for CAR in all recruited CRC patients, stage I–III CRC patients, and CRLM patients were 0.64, 0.63, and 0.33, respectively (Supplementary Figure 1).

### Correlation Between the Pretreatment C-Reactive Protein, Glasgow Prognostic Score, and C-Reactive Protein-to-Albumin Ratio and Clinicopathological Features

The correlation analysis in all CRC patients revealed that pretreatment CRP was positively correlated with sex, location of the primary tumor, pretreatment CEA and CA19-9, lymphatic invasion, and stage and negatively correlated with pathological differentiation. Pretreatment GPS was also revealed to be positively correlated with age, location of the primary tumor, pretreatment CEA and CA19-9, and stage and negatively correlated with pathological differentiation in CRC patients. In addition, pretreatment CAR was positively correlated with sex, location of the primary tumor, pretreatment CEA and CA19-9, and stage and negatively correlated with pathological differentiation in CRC patients (Table 1). In stage I–III CRC patients, the correlation analysis revealed that pretreatment CRP was positively correlated with sex and location of the primary tumor. Also, pretreatment GPS was positively correlated with age and pretreatment CEA. The analysis further revealed that pretreatment CAR was positively correlated with sex and the location of the primary tumor in stage I–III CRC patients (Table 2). The correlation analysis in CRLM patients revealed that pretreatment CRP was positively correlated with the location of the primary tumor, and pretreatment CEA and CA19-9. It was also revealed that pretreatment GPS was positively correlated with pretreatment CEA and CA19-9. The analysis also showed that pretreatment CAR was positively correlated with pretreatment CEA and CA19-9 in CRLM patients (Table 3).

**TABLE 1:** Significant correlations of pretreatment CRP, GPS, and CAR with the clinicopathological features in CRC patients.

**Parameter**	**Pretreatment CRP**		**Pretreatment GPS**		**Pretreatment CAR**	
	***r***	***p*-value**	***r***	***p*-value**	***r***	***p*-value**
Sex	0.173	**0.001**	0.042	0.414	0.169	**0.001**
Age	0.071	0.170	0.123	**0.017**	0.093	0.071
Location of primary tumor	0.211	**<0.001**	0.114	**0.026**	0.212	**<0.001**
Pathological differentiation	−0.122	**0.017**	−0.113	**0.028**	−0.122	**0.017**
Pretreatment CEA (ng/ml)	0.345	**<0.001**	0.215	**<0.001**	0.341	**<0.001**
Pretreatment CA19-9 (ng/ml)	0.310	**<0.001**	0.124	**0.015**	0.306	**<0.001**
Lymphatic invasion	0.082	0.110	0.149	**0.004**	0.084	0.101
Stage	0.247	**<0.001**	0.224	**<0.001**	0.261	**<0.001**

*If two variables are continuous, Pearson’s correlation analysis was used. If two variables are categorical, or one categorical and one continuous, Spearman’s correlation analysis was used. Values in bold indicate statistical significance (*p* < 0.05). CRP, C-reactive protein; GPS, Glasgow Prognostic Score; CAR, CRP-to-albumin ratio; CRC, colorectal cancer; CEA, carcinoembryonic antigen; CA19, carbohydrate antigen 19-9.*

**TABLE 2:** Significant correlations of pretreatment CRP, GPS, and CAR with the clinicopathological features of stage I–III CRC patients.

**Parameter**	**Pretreatment CRP**		**Pretreatment GPS**		**Pretreatment CAR**	
	***r***	***p*-value**	***r***	***p*-value**	***r***	***p*-value**
Sex	0.175	**0.002**	0.069	0.230	0.169	**0.003**
Age	−0.027	0.642	0.118	**0.039**	−0.002	0.974
Location of primary tumor	0.199	**<0.001**	0.093	0.104	0.204	**<0.001**
Pathological differentiation	−0.095	0.096	−0.056	0.331	−0.092	0.107
Pretreatment CEA (ng/ml)	−0.015	0.798	0.119	**0.037**	−0.013	0.817
Pretreatment CA19-9 (ng/ml)	0.047	0.408	0.013	0.822	0.049	0.397
Lymphatic invasion	−0.007	0.903	0.058	0.309	−0.013	0.814
Stage	−0.046	0.422	0.015	0.793	−0.052	0.363

*If two variables are continuous, Pearson’s correlation analysis was used. If there are two categorical variables, or one categorical and one continuous, Spearman’s correlation analysis was used. Values in bold indicate statistical significance (*p* < 0.05). CRP, C-reactive protein; GPS, Glasgow Prognostic Score; CAR, CRP-to-albumin ratio; CRC, colorectal cancer; CEA, carcinoembryonic antigen; CA19, carbohydrate antigen 19-9.*

**TABLE 3:** Significant correlations of pretreatment CRP, GPS, and CAR with the clinicopathological features of CRLM patients.

**Parameter**	**Pretreatment CRP**		**Pretreatment GPS**		**Pretreatment CAR**	
	***r***	***p*-value**	***r***	***p*-value**	***r***	***p*-value**
Sex	0.039	0.748	−0.094	0.431	0.035	0.774
Age	−0.068	0.568	0.106	0.373	0.093	0.071
Location of primary tumor	0.261	**0.027**	0.222	0.061	0.221	0.064
Pathological differentiation	−0.100	0.401	−0.142	0.233	−0.074	0.539
Pretreatment CEA (ng/ml)	0.409	**<0.001**	0.346	**0.003**	0.390	**<0.001**
Pretreatment CA19-9 (ng/ml)	0.367	**0.002**	0.380	**0.001**	0.349	**0.003**
Lymphatic invasion	−0.054	0.653	0.082	0.496	−0.019	0.876

*If two variables are continuous, Pearson’s correlation analysis was used. If there are two categorical variables, or one categorical and one continuous, Spearman’s correlation analysis was used. Values in bold indicate statistical significance (*p* < 0.05). CRP, C-reactive protein; GPS, Glasgow Prognostic Score; CAR, CRP-to-albumin ratio; CRLM, colorectal liver metastases; CEA, carcinoembryonic antigen; CA19, carbohydrate antigen 19-9.*

### Survival Analysis Based on Pretreatment Inflammatory Markers

For CRC patients, Kaplan–Meier analysis and the log-rank tests demonstrated that those with high CRP and GPS had poorer OS than did those with lower values of the corresponding indices. Kaplan–Meier analysis and log-rank tests also demonstrated that those with high CARs had poorer OS than did patients with lower corresponding values, both in the cohorts of stage I–III patients and CRLM patients (*p* < 0.05) (Supplementary Figures 2, 3).

**FIGURE 3:**
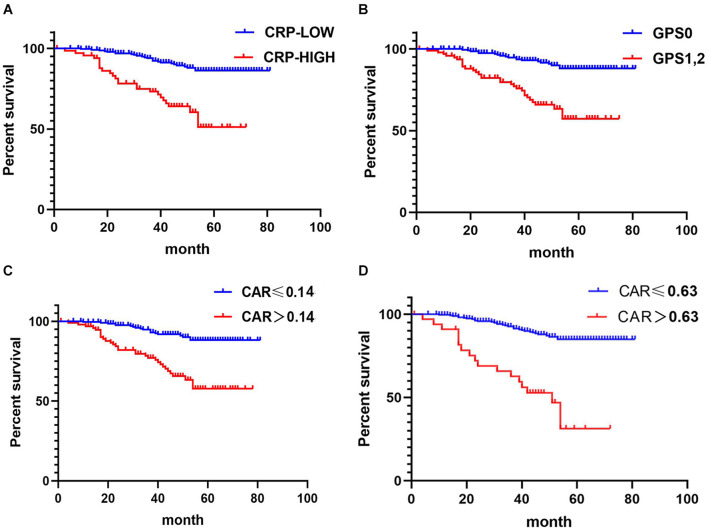
Relationship between C-reactive protein (CRP), CRP-to-albumin ratio (CAR), and Glasgow Prognostic Score (GPS) and overall survival (OS) in patients undergoing surgery for stage I–III colorectal cancer (CRC). **(A)** Relationship between the two CRP groups and OS. **(B)** Relationship between the two GPS groups and OS. **(C)** Relationship between the two CAR groups and OS. CAR was analyzed by grouping based on the cutoff value of the receiver operating characteristic (ROC) analysis in Figure 2B. **(D)** Relationship between the two CAR groups and OS. The optimal cutoff levels for CAR were produced using the X-tile plot in Supplementary Figure 1B.

Kaplan–Meier analysis and the log-rank tests also demonstrated that stage I–III CRC patients with high CRP, GPS, and CAR had poorer OS than those with lower values of the corresponding indices (*p* < 0.05) (Figure 3).

**FIGURE 4:**
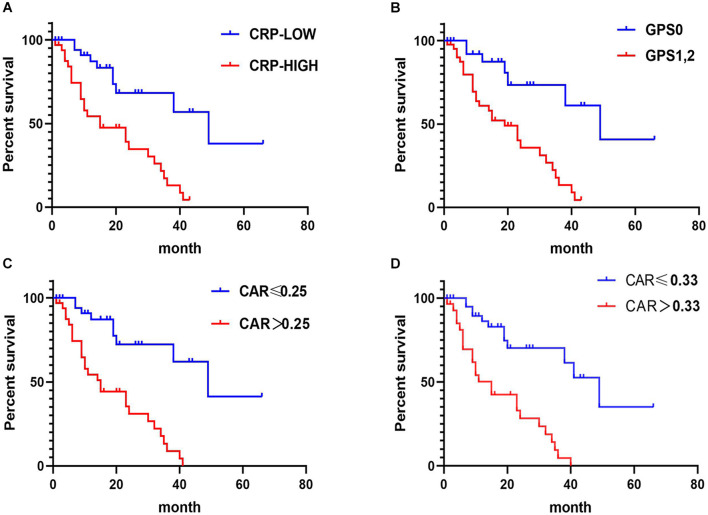
Relationship between C-reactive protein (CRP), CRP-to-albumin ratio (CAR), and Glasgow Prognostic Score (GPS) and overall survival (OS) in patients receiving treatment for colorectal liver metastases (CRLM). **(A)** Relationship between the two CRP groups and OS. **(B)** Relationship between the two GPS groups and OS. **(C)** Relationship between the two CAR groups and OS. CAR was analyzed by grouping based on the cutoff value of the receiver operating characteristic (ROC) analysis in Figure 2C. **(D)** Relationship between the two CAR groups and OS. The optimal cutoff levels for CAR were produced using the X-tile plot in Supplementary Figure 1C.

In CRLM patients, the results of Kaplan–Meier analysis and the log-rank tests showed that patients with high CRP, GPS, and CAR had poorer OS than those with corresponding lower values (*p* < 0.05) (Figure 4).

### Prognostic Value of Outcome Prediction Among the Three Inflammation-Based Prognostic Scores

**FIGURE 5:**
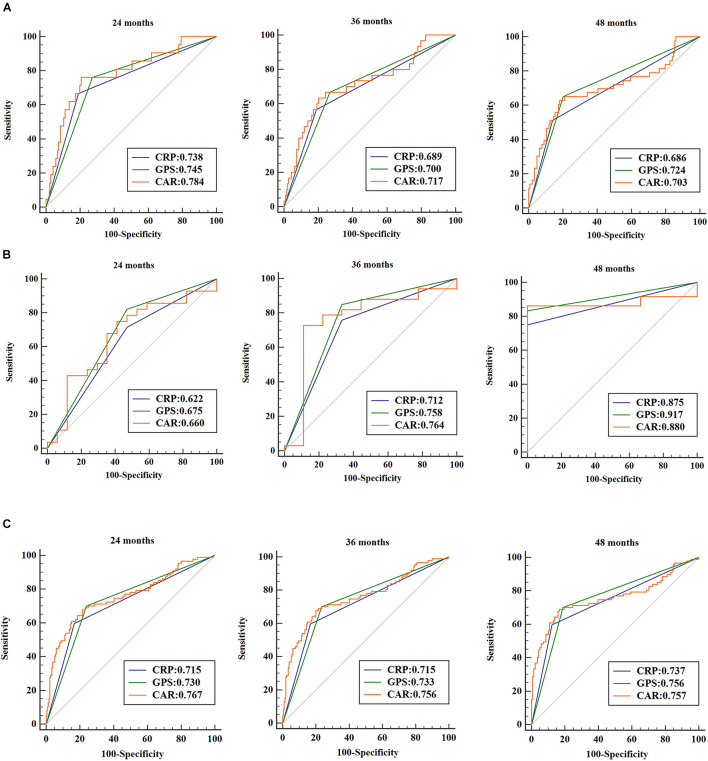
Receiver operating characteristic (ROC) curves were used to analyze patient survival at the 24-, 36-, and 48-month follow-up periods. The areas under the ROC curve (AUCs) assessed the discriminative ability of each inflammation-based prognostic score in stage I–III colorectal cancer (CRC) patients **(A)**, in colorectal liver metastasis (CRLM) patients **(B)**, and in all CRC patients **(C)**.

ROC curves were used to analyze patient survival at the 24-, 36-, and 48-month follow-up periods, and the AUC assessed the discriminative ability of each inflammation-based prognostic score in CRC patients, stage I–III CRC patients, and CRLM patients (Figure 5). Each prognostic score based on inflammation showed similar discriminative ability. These indicators show better performance in long-term prognostic value for CRLM patients than for stage I–III CRC patients.

### Prognostic Factors Influencing Long-Term Survival

The correlation values between OS and various clinicopathological factors in stage I–III CRC patients are shown in Table 4. In the univariate analysis, OS showed significant relationship with sex, location of the primary tumor, pathological differentiation, lymphatic invasion, pretreatment CEA, CRP, GPS, and CAR, and stage (all *p* < 0.05). Multivariate analysis indicated that the location of the primary tumor [hazard ratio (HR) = 0.3210, 95% CI = 0.164–0.627, *p* = 0.001], pathological differentiation (HR = 0.5014, 95% CI = 0.258–0.973, *p* = 0.041), pretreatment CEA (HR = 3.1573, 95% CI = 1.574–6.332, *p* = 0.001), and pretreatment CAR (HR = 3.9310, 95% CI = 2.116–7.303, *p* < 0.001) were independent prognostic factors for OS. Because CRP, CAR, and GPS contained the same data, three tables were made to consider them separately when performing a multifactor analysis. The HR of CAR was the highest; therefore, the table containing CAR was included in the main manuscript (Table 4); the rest of the results are presented as Supplementary Material (Supplementary Tables 1, 2).

**TABLE 4:** Correlations between overall survival and the clinicopathological factors in stage I–III CRC patients.

	**Univariate analysis**			**Multivariate analysis**		
	**Hazard ratio**	**95% CI**	***p*-value**	**Hazard ratio**	**95% CI**	***p*-value**
Sex (female *vs*. male)	2.0918	1.071–4.086	**0.031**	1.8118	0.913–3596	0.089
Age (≤ 60 *vs*. > 60 years)	1.6372	0.940–2.850	0.081			
Location of primary tumor (colon *vs*. rectum)	0.3523	0.187–0.663	**0.001**	0.3210	0.164–0.627	**0.001**
Pathological differentiation (poor *vs*. well, moderate)	0.4898	0.264–0.910	**0.024**	0.5014	0.258–0.973	**0.041**
Lymphatic invasion (ly0 *vs*. ly1, ly2, ly3)	3.2600	1.779–5.972	**<0.001**	1.5453	0.485–4.923	0.462
Pretreatment CEA (≤ 5 *vs*. > 5 ng/ml)	4.4961	2.302–8.782	**<0.001**	3.1573	1.574–6.332	**0.001**
Pretreatment CA19-9 (≤ 37 *vs*. > 37 ng/ml)	1.6682	0.834–3.337	0.148			
Pretreatment CRP (≤ 10 *vs*. > 10 ng/ml)	4.4098	2.527–7.696	**<0.001**			
Pretreatment GPS (0 *vs*. 1, 2)	4.6248	2.607–8.204	**<0.001**			
Pretreatment CAR (≤ 0.14 *vs*. > 0.14)	4.4661	2.520–7.917	**<0.001**	3.9310	2.116–7.303	**<0.001**
Stage (I, II *vs*. III)	2.6337	1.487–4.664	**<0.001**	1.6278	0.554–4.784	0.376

*Values in bold indicate statistical significance (*p* < 0.05). CRC, colorectal cancer; CEA, carcinoembryonic antigen; CA19, carbohydrate antigen 19-9.*

The correlations between OS and the clinicopathological factors in CRLM patients are shown in Table 5. In the univariate analysis, OS was significantly associated with pretreatment CA19-9, CRP, GPS, and CAR (all *p* < 0.05). Multivariate analysis indicated that pretreatment CAR (HR = 5.208, 95% CI = 2.189–12.386, *p* < 0.001) was an independent prognostic factor for OS. The HR of CAR was the highest; therefore, the table containing CAR was included in the main manuscript (Table 5); the rest of the results are presented as Supplementary Material (Supplementary Tables 3, 4).

**TABLE 5:** Correlations between overall survival and the clinicopathological factors in CRLM patients.

	**Univariate analysis**			**Multivariate analysis**		
	**Hazard ratio**	**95% CI**	***p*-value**	**Hazard ratio**	**95% CI**	***p*-value**
Sex (female *vs*. male)	0.7009	0.342–1.438	0.332			
Age (≤ 60 *vs*. > 60 years)	1.6772	0.863–3.258	0.127			
Location of primary tumor (colon *vs*. rectum)	0.9722	0.506–1.866	0.932			
Pathological differentiation (poor *vs*. well, moderate)	1.0564	0.461–2.420	0.897			
Lymphatic invasion (ly0 *vs*. ly1, ly2, ly3)	1.9437	0.465–8.125	0.362			
Pretreatment CEA (≤ 5 *vs*. > 5 ng/ml)	1.0035	0.457–2.203	0.993			
Pretreatment CA19-9 (≤ 37 *vs*. > 37 ng/ml)	2.1406	1.087–4.215	**0.028**	1.0387	0.503–2.144	0.918
Pretreatment CRP (≤ 10 *vs*. > 10 ng/ml)	3.8803	2.818–8.282	**<0.001**			
Pretreatment GPS (0 *vs*. 1, 2)	4.5748	1.889–11.08	**<0.001**			
Pretreatment CAR (≤ 0.25 *vs*. > 0.25)	5.3006	2.388–11.77	**<0.001**	5.2076	2.189–12.386	**<0.001**

*Values in bold indicate statistical significance (*p* < 0.05). CRLM, colorectal liver metastases; CEA, carcinoembryonic antigen; CA19, carbohydrate antigen 19-9; CRP, C-reactive protein; GPS, Glasgow Prognostic Score; CAR, CRP-to-albumin ratio.*

The correlations between OS and the clinicopathological factors in CRC patients are shown in Supplementary Table 5. In the univariate analysis, OS showed significant relationship with sex, location of the primary tumor, pathological differentiation, lymphatic invasion, pretreatment CEA, CRP, GPS, and CAR, and stage (all *p* < 0.05). Multivariate analysis indicated that the location of the primary tumor (HR = 0.5773, 95% CI = 0.367–0.909, *p* = 0.018), lymphatic invasion (HR = 2.9439, 95% CI = 1.664–5.208, *p* < 0.001), pretreatment CEA (HR = 2.2166, 95% CI = 1.304–3.768, *p* = 0.003), stage (HR = 3.4852, 95% CI = 2.069–5.872, *p* < 0.001), and pretreatment CAR (HR = 4.3122, 95% CI = 2.647–7.024, *p* < 0.001) were independent prognostic factors for OS. The HR of CAR was the highest; the table containing CAR, CRP, GPS are presented as Supplementary Material (Supplementary Tables 5–7).

### Establishing Colorectal Cancer Survival Nomograms Based on the Prognostic Scores of Inflammation

**FIGURE 6:**
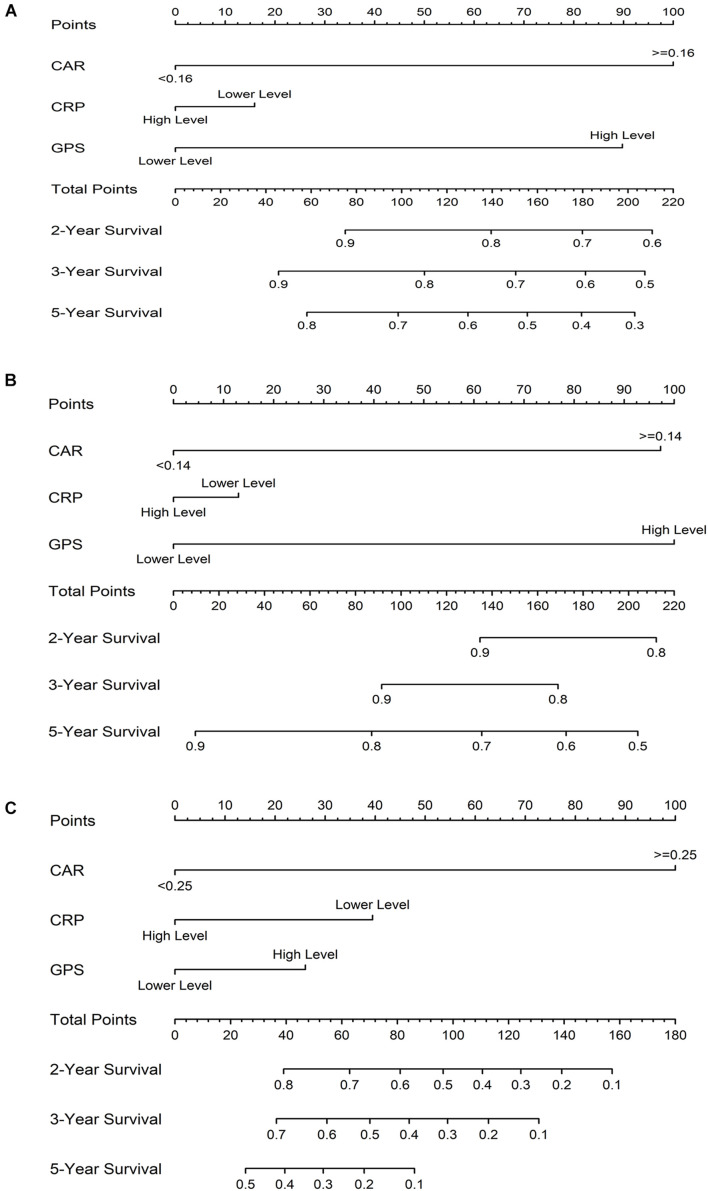
Prognostic nomograms for overall survival (OS) based on the prognostic scores of inflammation in colorectal cancer (CRC) patients **(A)**, in the stage I–III CRC cohort **(B)**, and in the colorectal liver metastases (CRLM) cohort **(C)**.

The prognostic nomogram for OS in CRC patients is shown in Figure 6A. The *C*-index for OS prediction was 0.85 (95% CI = 0.80–0.91). Figure 6B shows the prognostic nomogram for OS in the stage I–III CRC cohort, with a *C*-index for OS prediction of 0.81 (95% CI = 0.73–0.88). For the CRLM cohort, the prognostic nomogram for OS is shown in Figure 6C. The *C*-index for OS prediction was 0.81 (95% CI = 0.70–0.93). The values of the *C*-index suggest that the nomograms based on the prognostic scores of inflammation were useful predictors for survival of CRC patients. The calibration plot demonstrated a favorable agreement between the predicted and the observed values (Supplementary Figure 4).

## Discussion

CRC is a major health problem worldwide, with 1.8 million new cases and 881,000 deaths a year, accounting for approximately one-tenth of all new cancer cases and deaths (Bray et al., 2018). Over the past 20 years, the United States and Japan have identified patients with early-stage rectal cancer using various examination methods and intervened with the disease as early as possible, effectively reducing the incidence of CRC and the associated mortality (Schreuders et al., 2015). Therefore, effective early diagnosis has an important effect on patient prognosis. Currently, the CEA level is widely used as a prognostic biomarker. However, the sensitivity and specificity of this method are limited in the prognosis of CRC. The identification of new tumor biomarkers is essential to improve the diagnosis and survival of patients.

There is increasing evidence that cancer is related to inflammation. The link between inflammation and cancer is generally such that tumors usually appear in sites of chronic inflammation, and inflammatory cells can be found in tumor biopsy samples (Kinoshita et al., 2013). In addition, recent studies have shown that inflammation was closely related to cancer progression and metastasis. This is probably because the growth or invasion of tumors can cause tissue inflammation, and the production of inflammatory cytokines can, in turn, promote persistent tumor growth, invasion, and metastasis. Furthermore, some studies even suggested that the inflammatory microenvironment could be the seventh sign of cancer (Balkwill and Mantovani, 2001; Qian et al., 2011; Elinav et al., 2013). Therefore, inflammatory markers can predict the prognosis of patients with various cancers, including CRC.

CRP, an inflammatory marker, is an acute-phase reactant synthesized by liver cells (Morris-Stiff et al., 2008). An elevated CRP level reflects the inflammatory response caused by tumor necrosis. Formation of the inflammatory microenvironment is beneficial for persistent tumor growth, invasion, and metastasis (Wong et al., 2007). In this study, CRLM patients had higher CRP levels, which supported this notion, to some extent. Albumin also participates in inflammation. Low albumin levels are associated with poor long-term survival rates in various types of cancer and are more common in patients with advanced cancer (Wang et al., 2019). The prognostic scores of GPS and CAR are based on the CRP and albumin levels, and they may be related to inflammation and poor survival. In the past few years, CRP, GPS, and CAR have been shown to have prognostic value in patients with hepatocellular carcinoma (Kinoshita et al., 2015) and gallbladder (Utsumi et al., 2020), pancreatic (Primavesi et al., 2020), ovarian (Liu et al., 2017), esophageal (Sakai et al., 2020), and gastric (Okugawa et al., 2020) cancers.

This study is the first that directly compares the prognostic value of CRP, GPS, and CAR in patients with primary CRC and liver metastases over multiple time periods. It is interesting to note that these indicators showed better performance in long-term prognostic value for CRLM patients than for stage I–III CRC patients. Pretreatment CRP, GPS, and CAR were independently associated with OS in CRC patients, stage I–III CRC patients, and CRLM patients. Pretreatment CEA was independently associated with the OS of CRC patients and stage I–III CRC patients, while there was no significant relationship between CEA and OS in CRLM patients. However, it is common for CRLM patients to have normal CEA levels. One study showed that approximately 55% (271/491) of CRC patients undergoing surgery had normal preoperative serum CEA levels (Ishizuka et al., 2012). The above evidence shows that, compared with tumor markers, inflammation-based prognostic systems demonstrate a unique predictive capacity in CRC patients.

Previous studies have reported that the peripheral inflammation markers, including the levels of CRP, GPS, and CAR, are related to the prognosis of CRC patients (Ishizuka et al., 2016; Chen et al., 2017), and this was confirmed in our study. However, results of the subgroup analysis of patients with primary CRC and patients with metastases in the same study have not been reported. As we all know, distant metastasis is one of the important factors affecting the prognosis of patients. Therefore, the results are biased when the analysis for OS was performed on stage I–III patients and patients with distant metastases as a group. Even in studies that have analyzed stage I–III CRC patients or metastatic patients alone (Shibutani et al., 2016a,b; Sakamoto et al., 2020; Son et al., 2020), the optimal cutoff levels of CAR and the ethnicity have been different. Therefore, the prognostic value of CAR in patients with CRC cannot be clearly compared between different studies. Racial differences among patients may also contribute to the differences, but the reasons for the differences in outcomes remain unclear. In this study, we tried to ensure the consistency of the study participants in order to explore the most suitable beneficiary group. Patients with stage I–III CRC and patients with liver metastases were discussed separately. This study is helpful for clinicians to detect patients with poor prognosis early and intervene in time so as to prolong their survival time.

Presently, the commonly used methods to determine the optimal cutoff values included the median value method, ROC analysis, and the X-tile software. In this study, in addition to obtaining the optimal cutoff value of CAR through ROC analysis for subsequent analysis, we also used X-tile software to obtain the optimal cutoff value. Kaplan–Meier analysis and the log-rank tests showed that, patients with high CAR values obtained by the two methods have worse OS than those with low values, which scientifically reveals the significance of CAR in the prognosis of CRC patients, both in the stage I–III and CRLM patient cohorts. However, the optimal cutoff value obtained using the X-tile software for stage I–III CRC patients was higher than that for CRLM patients, which is not conducive to clinical application. This may be because there are not enough CRLM patients included.

In this study, CAR was able to accurately divide CRC patients into two independent groups, similarly to CRP and GPS. Because CAR and GPS are based on only two measured parameters and have high AUC values, they can be conventionally available, simple, and inexpensive. Some studies have found that inflammation markers were related to tumor progression (Xu et al., 2015; Ishizuka et al., 2016). We also found that pretreatment CRP, GPS, and CAR were significantly correlated with stage in CRC patients. Therefore, it is possible to determine the optimal cutoff value of CAR for tumors at different stages. However, the best cutoff value for CAR reported in previous studies was different from that obtained in this study, and there was a big gap in the CAR obtained in this study. An accurate determination of the optimal cutoff value of CAR to facilitate the promotion and application of this prognostic index in clinical practice requires further research. GPS is a cumulative scoring method based on a normal reference range. The simplicity and consistency of this indicator makes it very practical for clinical use. Climent et al. (2019) found that the OS difference between the high and low CAR groups was not significant, which is inconsistent with our results. This may be because their research subject was mismatch repair-deficient CRC. To improve the accuracy of prognosis, we have developed survival nomograms based on the three markers, which might increase the clinical significance of the study. The nomograms performed well in predicting survival, and predictions were supported by the *C*-index (0.85, 0.81, and 0.81 for CRC patients, stage I–III CRC cohort, and the CRLM cohort, respectively) and the calibration plot.

There are some potential limitations to this study. Firstly, this is a retrospective single-center study. Secondly, the same variables (CRP and albumin) were included in the analysis of CRP, GPS, and CAR in our multivariate analysis. Thirdly, the best cutoff value for pretreatment CAR is unknown. Therefore, a large-scale, multicenter prospective study is still necessary in the future.

In summary, CRC patients with high CRP, GPS and CAR values have worse OS than those with corresponding low values. The three markers have independent prognostic values in patients with CRC, both in the cohorts of stage I–III patients and CRLM patients. Furthermore, we have developed survival nomograms based on the three markers, which might increase the clinical significance of the study.

## Data Availability Statement

The datasets presented in this study can be found in online repositories. The names of the repository/repositories and accession number(s) can be found in the article/Supplementary Material.

## Author Contributions

JZ designed the study, performed the statistical analysis, and prepared the manuscript. JZ, WW, SN, and BH acquired the data. JL helped with quality control of data and algorithms. JZ and JL analyzed and interpreted the data. KL and LZ edited the manuscript. All authors reviewed the manuscript and approved the final version.

## Conflict of Interest

The authors declare that the research was conducted in the absence of any commercial or financial relationships that could be construed as a potential conflict of interest.

## Publisher’s Note

All claims expressed in this article are solely those of the authors and do not necessarily represent those of their affiliated organizations, or those of the publisher, the editors and the reviewers. Any product that may be evaluated in this article, or claim that may be made by its manufacturer, is not guaranteed or endorsed by the publisher.

## Abbreviations

CRC: colorectal cancer
CRLM: colorectal liver metastases
CEA: carcinoembryonic antigen
CA19-9: carbohydrate antigen 19-9
CAR: CRP/albumin ratio
CRP: C-reactive protein
GPS: Glasgow prognostic score
ROC: receiver operating characteristic
AUC: area under the ROC curve
CI: confidence interval.
