# Supplementary material for: Prognostic Value of C-Reactive Protein, Glasgow Prognostic Score, and C-Reactive Protein-to-Albumin Ratio in Colorectal Cancer
Source: Front Cell Dev Biol. 2021 Oct 26;9:637650. doi: 10.3389/fcell.2021.637650 (PMC8577830; doi:10.3389/fcell.2021.637650)
Supplement: Supplementary file 1 [file Data_Sheet_1.docx]

**Supplementary Table 1** The correlations between overall survival and various clinicopathological factors in stage Ⅰ-III CRC patients

|  | **Univariate analysis** | | | **Multivariate analysis** | | |
| --- | --- | --- | --- | --- | --- | --- |
|  | **Hazard ratio** | **95 % CI** | ***p* value** | **Hazard ratio** | **95 % CI** | ***p* value** |
| Sex(female vs. male) | 2.0918 | 1.071-4.086 | **0.031** | 1.7338 | 0.867-3.466 | 0.120 |
| Age (≤60 vs. ＞60) | 1.6372 | 0.940-2.850 | 0.081 |  |  |  |
| Location of primary tumor (colon vs. rectum) | 0.3523 | 0.187-0.663 | **0.001** | 0.3373 | 0.174-0.653 | **0.001** |
| Pathological differentiation (poorly vs. well, moderately) | 0.4898 | 0.264-0.910 | **0.024** | 0.5428 | 0.277-1.066 | 0.076 |
| Lymphatic invasion (ly 0 vs. ly1, 2, 3) | 3.2600 | 1.779-5.972 | **<0.001** | 1.8990 | 0.597-6.044 | 0.278 |
| Pretreatment CEA (≤5 ng/mL vs. >5 ng/mL) | 4.4961 | 2.302-8.782 | **<0.001** | 3.4383 | 1.727-6.846 | **<0.001** |
| Pretreatment CA19-9 (≤37 ng/ml vs. >37 ng/ml) | 1.6682 | 0.834-3.337 | 0.148 |  |  |  |
| Pretreatment CRP(≤10ng/mL vs. >10 ng/mL) | 4.4098 | 2.527-7.696 | **<0.001** | 3.7190 | 2.0174-6.856 | **<0.001** |
| Pretreatment GPS (0 vs. 1,2) | 4.6248 | 2.607-8.204 | **<0.001** |  |  |  |
| Pretreatment CAR (≤0.14 vs. > 0.14) | 4.4661 | 2.520-7.917 | **<0.001** |  |  |  |
| Stage (I, II vs. III) | 2.6337 | 1.487-4.664 | **<0.001** | 1.3597 | 0.461-4.007 | 0.5774 |

**Supplementary Table 2** The correlations between overall survival and various clinicopathological factors in stage Ⅰ-III CRC patients

|  | **Univariate analysis** | | | **Multivariate analysis** | | |
| --- | --- | --- | --- | --- | --- | --- |
|  | **Hazard ratio** | **95 % CI** | ***p* value** | **Hazard ratio** | **95 % CI** | ***p* value** |
| Sex(female vs. male) | 2.0918 | 1.071-4.086 | **0.031** | 1.9846 | 1.003-3.929 | **0.049** |
| Age (≤60 vs. ＞60) | 1.6372 | 0.940-2.850 | 0.081 |  |  |  |
| Location of primary tumor (colon vs. rectum) | 0.3523 | 0.187-0.663 | **0.001** | 0.3630 | 0.188-0.701 | **0.003** |
| Pathological differentiation (poorly vs. well, moderately) | 0.4898 | 0.264-0.910 | **0.024** | 0.4860 | 0.251-0.941 | **0.032** |
| Lymphatic invasion (ly 0 vs. ly1, 2, 3) | 3.2600 | 1.779-5.972 | **<0.001** | 1.9106 | 0.606-6.025 | 0.269 |
| Pretreatment CEA (≤5 ng/mL vs. >5 ng/mL) | 4.4961 | 2.302-8.782 | **<0.001** | 3.6890 | 1.852-7.349 | **<0.001** |
| Pretreatment CA19-9 (≤37 ng/ml vs. >37 ng/ml) | 1.6682 | 0.834-3.337 | 0.148 |  |  |  |
| Pretreatment CRP(≤10ng/mL vs. >10 ng/mL) | 4.4098 | 2.527-7.696 | **<0.001** |  |  |  |
| Pretreatment GPS (0 vs. 1,2) | 4.6248 | 2.607-8.204 | **<0.001** | 3.8907 | 2.140-7.074 | **<0.001** |
| Pretreatment CAR (≤0.14 vs. > 0.14) | 4.4661 | 2.520-7.917 | **<0.001** |  |  |  |
| Stage (I, II vs. III) | 2.6337 | 1.487-4.664 | **<0.001** | 1.2247 | 0.422-3.557 | 0.709 |

**Supplementary Table 3** The correlations between overall survival and various clinicopathological factors in CRLM patients

|  | **Univariate analysis** | | | **Multivariate analysis** | | |
| --- | --- | --- | --- | --- | --- | --- |
|  | **Hazard ratio** | **95 % CI** | ***p* value** | **Hazard ratio** | **95 % CI** | ***p* value** |
| Sex(female vs. male) | 0.7009 | 0.342-1.438 | 0.332 |  |  |  |
| Age (≤60 vs. ＞60) | 1.6772 | 0.863-3.258 | 0.127 |  |  |  |
| Location of primary tumor (colon vs. rectum) | 0.9722 | 0.506-1.866 | 0.932 |  |  |  |
| Pathological differentiation (poorly vs. well, moderately) | 1.0564 | 0.461-2.420 | 0.897 |  |  |  |
| Lymphatic invasion (ly 0 vs. ly1, 2, 3) | 1.9437 | 0.465-8.125 | 0.362 |  |  |  |
| Pretreatment CEA (≤5 ng/mL vs. >5 ng/mL) | 1.0035 | 0.457-2.203 | 0.993 |  |  |  |
| Pretreatment CA19-9 (≤37 ng/ml vs. >37 ng/ml) | 2.1406 | 1.087-4.215 | **0.028** | 1.2714 | 0.615-2.630 | 0.517 |
| Pretreatment CRP(≤10ng/mL vs. >10 ng/mL) | 3.8803 | 2.818-8.282 | **<0.001** | 3.5116 | 1.549-7.961 | **0.003** |
| Pretreatment GPS (0 vs. 1,2) | 4.5748 | 1.889-11.08 | **<0.001** |  |  |  |
| Pretreatment CAR (≤0.25 vs. > 0.25) | 5.3006 | 2.388-11.77 | **<0.001** |  |  |  |

**Supplementary Table 4** The correlations between overall survival and various clinicopathological factors in CRLM patients

|  | **Univariate analysis** | | | **Multivariate analysis** | | |
| --- | --- | --- | --- | --- | --- | --- |
|  | **Hazard ratio** | **95 % CI** | ***p* value** | **Hazard ratio** | **95 % CI** | ***p* value** |
| Sex(female vs. male) | 0.7009 | 0.342-1.438 | 0.332 |  |  |  |
| Age (≤60 vs. ＞60) | 1.6772 | 0.863-3.258 | 0.127 |  |  |  |
| Location of primary tumor (colon vs. rectum) | 0.9722 | 0.506-1.866 | 0.932 |  |  |  |
| Pathological differentiation (poorly vs. well, moderately) | 1.0564 | 0.461-2.420 | 0.897 |  |  |  |
| Lymphatic invasion (ly 0 vs. ly1, 2, 3) | 1.9437 | 0.465-8.125 | 0.362 |  |  |  |
| Pretreatment CEA (≤5 ng/mL vs. >5 ng/mL) | 1.0035 | 0.457-2.203 | 0.993 |  |  |  |
| Pretreatment CA19-9 (≤37 ng/ml vs. >37 ng/ml) | 2.1406 | 1.087-4.215 | **0.028** | 1.2847 | 0.633-2.609 | 0.488 |
| Pretreatment CRP(≤10ng/mL vs. >10 ng/mL) | 3.8803 | 2.818-8.282 | **<0.001** |  |  |  |
| Pretreatment GPS (0 vs. 1,2) | 4.5748 | 1.889-11.08 | **<0.001** | 4.1241 | 1.619-10.503 | **0.003** |
| Pretreatment CAR (≤0.25 vs. > 0.25) | 5.3006 | 2.388-11.77 | **<0.001** |  |  |  |

**Supplementary Table 5** The correlations between overall survival and various clinicopathological factors in CRC patients

|  | **Univariate analysis** | | | **Multivariate analysis** | | |
| --- | --- | --- | --- | --- | --- | --- |
|  | **Hazard ratio** | **95 % CI** | ***p*-value** | **Hazard ratio** | **95 % CI** | ***p*-value** |
| Sex(female vs. male) | 1.6344 | 1.007-2.652 | **0.047** | 1.1662 | 0.706-1.928 | 0.549 |
| Age (≤60 vs. ＞60) | 1.4766 | 0.969-2.250 | 0.070 |  |  |  |
| Location of primary tumor (colon vs. rectum) | 0.5167 | 0.331-0.807 | **0.004** | 0.5773 | 0.367-0.909 | **0.018** |
| Pathological differentiation (poorly vs. well, moderately) | 0.6036 | 0.369-0.987 | **0.044** | 1.0446 | 0.625-1.747 | 0.868 |
| Lymphatic invasion (ly 0 vs. ly1, 2, 3) | 4.8003 | 2.821-8.168 | **<0.001** | 2.9439 | 1.664-5.208 | **<0.001** |
| Pretreatment CEA (≤5 ng/mL vs. >5 ng/mL) | 3.964 | 2.381-6.559 | **<0.001** | 2.2166 | 1.304-3.768 | **0.003** |
| Pretreatment CA19-9 (≤37 ng/ml vs. >37 ng/ml) | 2.8181 | 1.800-4.412 | **<0.001** | 1.4298 | 0.861-2.376 | 0.168 |
| Pretreatment CRP(≤10ng/mL vs. >10 ng/mL) | 5.6080 | 3.643-8.634 | **<0.001** |  |  |  |
| Pretreatment GPS (0 vs. 1,2) | 5.7895 | 3.648-9.188 | **<0.001** |  |  |  |
| Pretreatment CAR (≤0.16 vs. > 0.16) | 6.1610 | 3.918-9.688 | **<0.001** | 4.3122 | 2.647-7.024 | **<0.001** |
| Stage (I, II, III vs CRLM.) | 10.019 | 6.348-15.810 | **<0.001** | 3.4852 | 2.069-5.872 | **<0.001** |

**Supplementary Table 6** The correlations between overall survival and various clinicopathological factors in CRC patients

|  | **Univariate analysis** | | | **Multivariate analysis** | | |
| --- | --- | --- | --- | --- | --- | --- |
|  | **Hazard ratio** | **95 % CI** | ***p*-value** | **Hazard ratio** | **95 % CI** | ***p*-value** |
| Sex(female vs. male) | 1.6344 | 1.007-2.652 | **0.047** | 1.1221 | 0.677-1.860 | 0.655 |
| Age (≤60 vs. ＞60) | 1.4766 | 0.969-2.250 | 0.070 |  |  |  |
| Location of primary tumor (colon vs. rectum) | 0.5167 | 0.331-0.807 | **0.004** | 0.6453 | 0.408-1.020 | 0.061 |
| Pathological differentiation (poorly vs. well, moderately) | 0.6036 | 0.369-0.987 | **0.044** | 1.0568 | 0.630-1.774 | 0.834 |
| Lymphatic invasion (ly 0 vs. ly1, 2, 3) | 4.8003 | 2.821-8.168 | **<0.001** | 3.0306 | 1.716-5.353 | **<0.001** |
| Pretreatment CEA (≤5 ng/mL vs. >5 ng/mL) | 3.964 | 2.381-6.559 | **<0.001** | 2.3726 | 1.399-4.022 | **0.001** |
| Pretreatment CA19-9 (≤37 ng/ml vs. >37 ng/ml) | 2.8181 | 1.800-4.412 | **<0.001** | 1.3139 | 0.788-2.190 | 0.295 |
| Pretreatment CRP(≤10ng/mL vs. >10 ng/mL) | 5.6080 | 3.643-8.634 | **<0.001** | 3.8314 | 2.383-6.160 | **<0.001** |
| Pretreatment GPS (0 vs. 1,2) | 5.7895 | 3.648-9.188 | **<0.001** |  |  |  |
| Pretreatment CAR (≤0.16 vs. > 0.16) | 6.1610 | 3.918-9.688 | **<0.001** |  |  |  |
| Stage (I, II, III vs CRLM.) | 10.019 | 6.348-15.810 | **<0.001** | 3.7048 | 2.200-6.240 | **<0.001** |

**Supplementary Table 7** The correlations between overall survival and various clinicopathological factors in CRC patients

|  | **Univariate analysis** | | | **Multivariate analysis** | | |
| --- | --- | --- | --- | --- | --- | --- |
|  | **Hazard ratio** | **95 % CI** | ***p*-value** | **Hazard ratio** | **95 % CI** | ***p*-value** |
| Sex(female vs. male) | 1.6344 | 1.007-2.652 | **0.047** | 1.3256 | 0.806-2.181 | 0.267 |
| Age (≤60 vs. ＞60) | 1.4766 | 0.969-2.250 | 0.070 |  |  |  |
| Location of primary tumor (colon vs. rectum) | 0.5167 | 0.331-0.807 | **0.004** | 0.6486 | 0.409-1.028 | 0.065 |
| Pathological differentiation (poorly vs. well, moderately) | 0.6036 | 0.369-0.987 | **0.044** | 0.9520 | 0.571-1.588 | 0.850 |
| Lymphatic invasion (ly 0 vs. ly1, 2, 3) | 4.8003 | 2.821-8.168 | **<0.001** | 2.7892 | 1.580-4.926 | **<0.001** |
| Pretreatment CEA (≤5 ng/mL vs. >5 ng/mL) | 3.964 | 2.381-6.559 | **<0.001** | 2.4207 | 1.426-4.110 | **0.001** |
| Pretreatment CA19-9 (≤37 ng/ml vs. >37 ng/ml) | 2.8181 | 1.800-4.412 | **<0.001** | 1.3328 | 0.799-2.224 | 0.272 |
| Pretreatment CRP(≤10ng/mL vs. >10 ng/mL) | 5.6080 | 3.643-8.634 | **<0.001** |  |  |  |
| Pretreatment GPS (0 vs. 1,2) | 5.7895 | 3.648-9.188 | **<0.001** | 4.0043 | 2.461-6.516 | **<0.001** |
| Pretreatment CAR (≤0.16 vs. > 0.16) | 6.1610 | 3.918-9.688 | **<0.001** |  |  |  |
| Stage (I, II, III vs CRLM.) | 10.019 | 6.348-15.810 | **<0.001** | 3.8795 | 2.302-6.538 | **<0.001** |


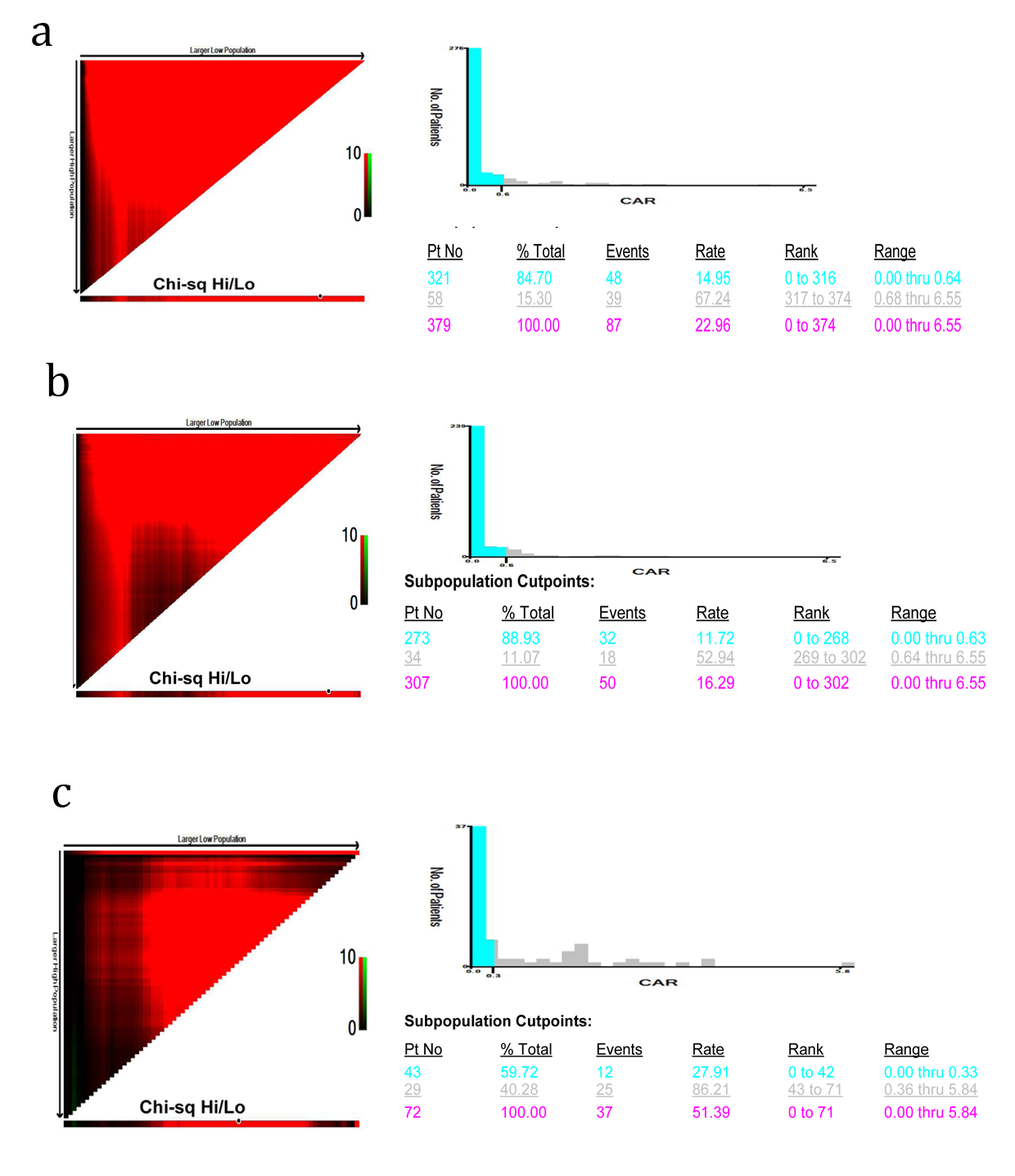


**Supplementary Figure 1.** **Optimal cutoff levels for CAR were produced by X-tile plot** **a** in all recruited CRC patients; **b** in all CRC patients with stage I–III; **c** in CRLM patients.

**
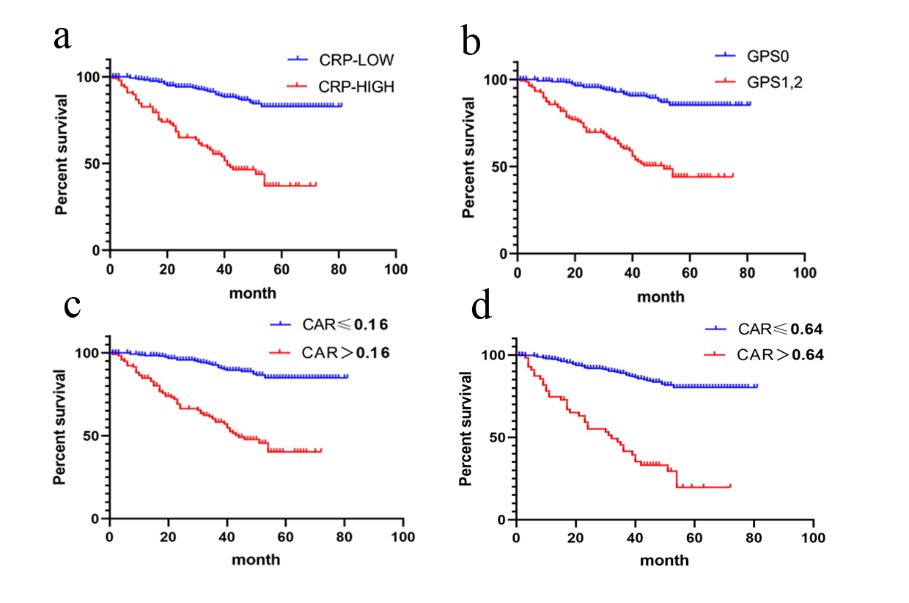
**

**Supplementary Figure 2.** **Relationship between CRP, CAR, GPS and overall survival in patients receiving treatment for CRC patients.** **a** Relationship between the two CRP groups and overall survival. **b** Relationship between the two GPS groups and overall survival. **c** Relationship between the two CAR groups and overall survival. CAR was analyzed by grouping based on the cutoff value of ROC analysis in Figure 2a. d Relationship between the two CAR groups and overall survival. Optimal cutoff levels for CAR were produced by X-tile plot in Supplementary Figure 1a.


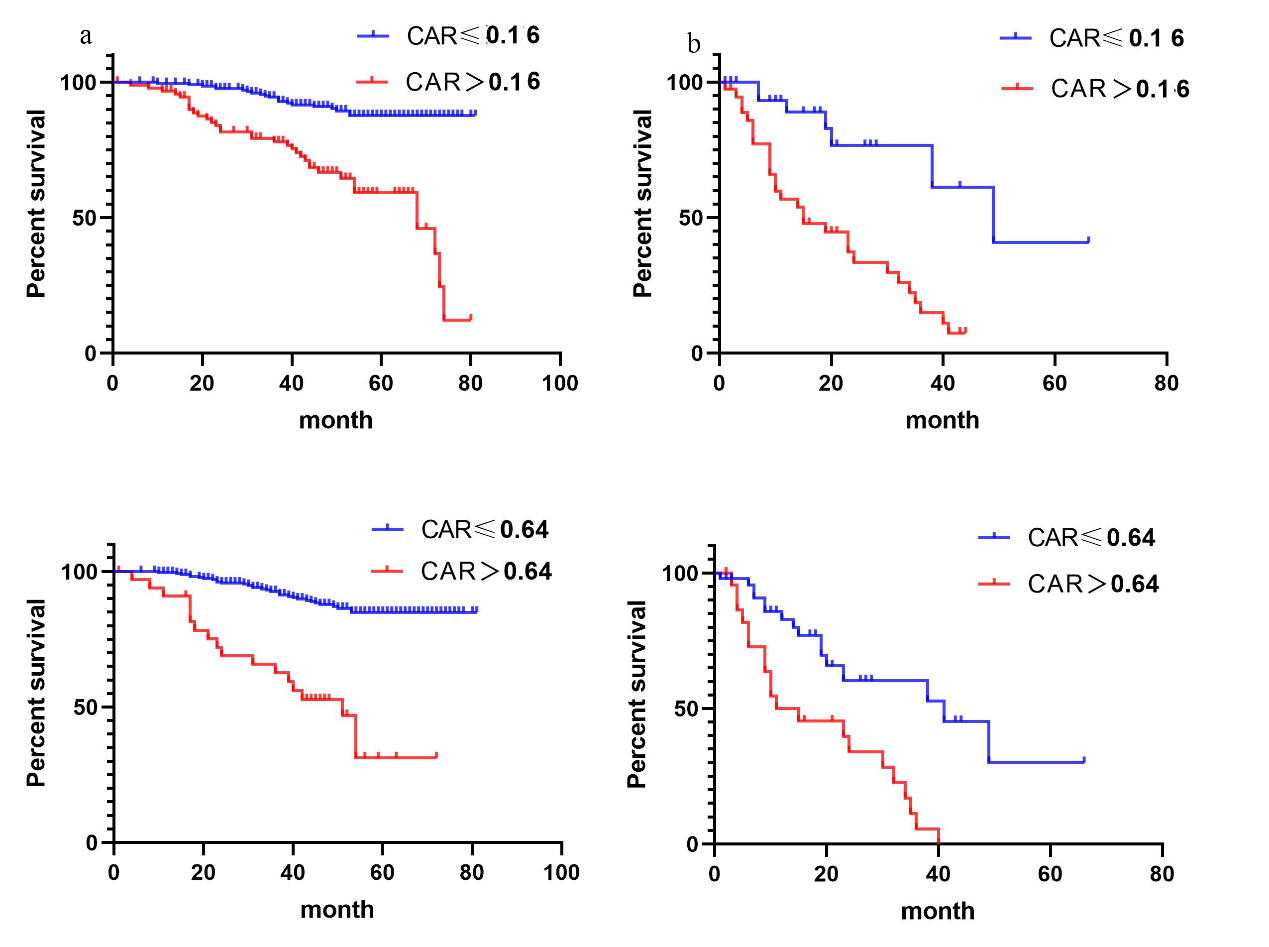


**Supplementary Figure 3.** **Relationship between CAR and overall survival in patients receiving treatment** **for stage I–III CRC patients and CRLM patients.** **a** Relationship between the two CAR groups and overall survival for stage I–III CRC. CAR was analyzed by grouping based on the cutoff value of ROC analysis in Figure 2a. **b** Relationship between the two CAR groups and overall survival for CRLM patients. CAR was analyzed by grouping based on the cutoff value of ROC analysis in Figure 2a. **c** Relationship between the two CAR groups and overall survival for stage I–III CRC. Optimal cutoff levels for CAR were produced by X-tile plot in Supplementary Figure 1a. **d** Relationship between the two CAR groups and overall survival for CRLM patients. Optimal cutoff levels for CAR were produced by X-tile plot in Supplementary Figure 1a.


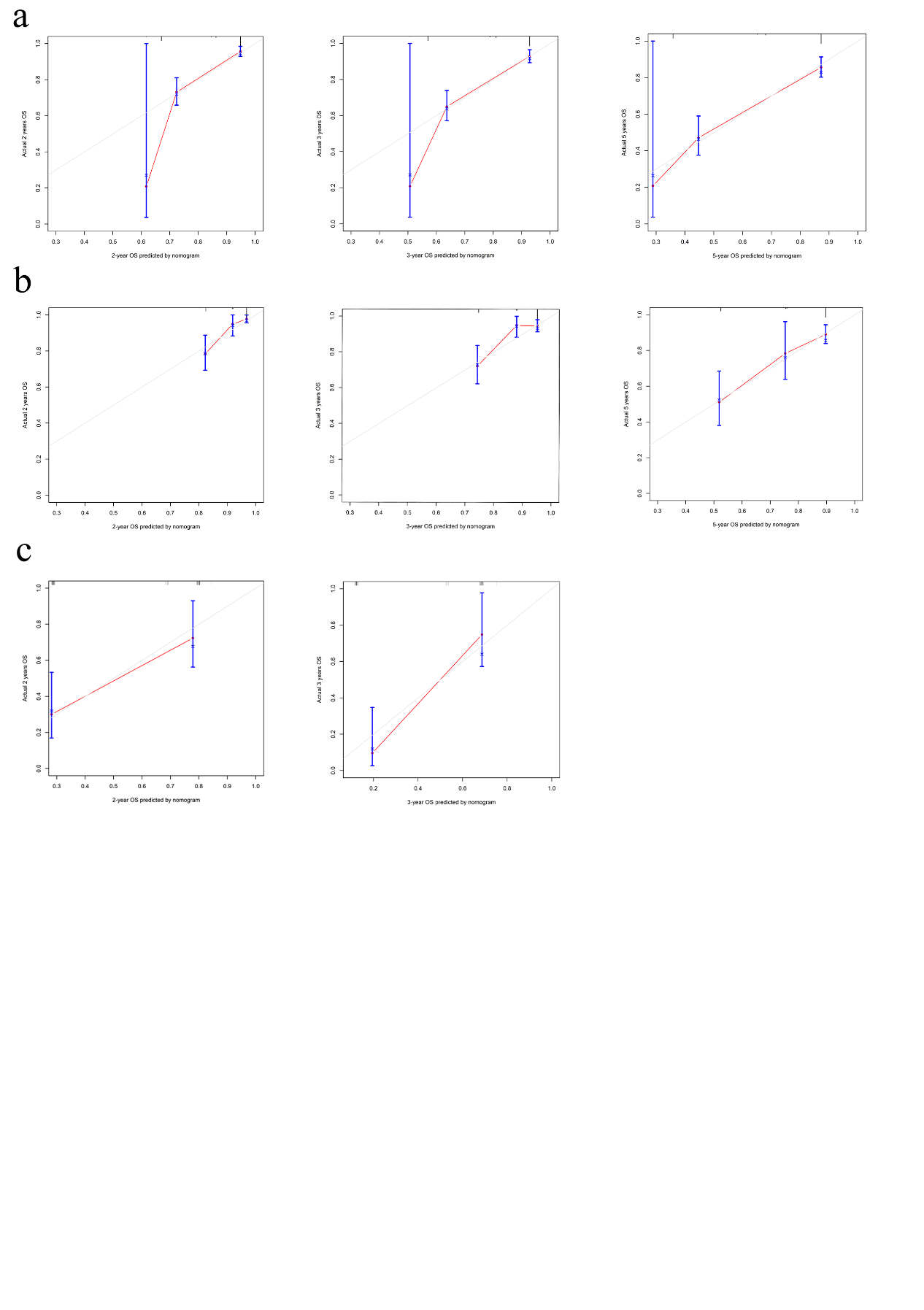


**Supplementary Figure 4. The calibration curve for predicting patient survival at 2 years, 3 years and 5 years a** in CRC patients **b** in the stage I–III CRC cohort **c** in the CRLM cohort. Nomogram-predicted probability of overall survival is plotted on the x-axis; actual overall survival is plotted on the y-axis
